# Supplementary material for: NFAT2 is a critical regulator of the anergic phenotype in chronic lymphocytic leukaemia
Source: Nat Commun. 2017 Oct 2;8:755. doi: 10.1038/s41467-017-00830-y (PMC5624906; doi:10.1038/s41467-017-00830-y)
Supplement: Supplementary file 1 — Supplementary Information [file 41467_2017_830_MOESM1_ESM.pdf]

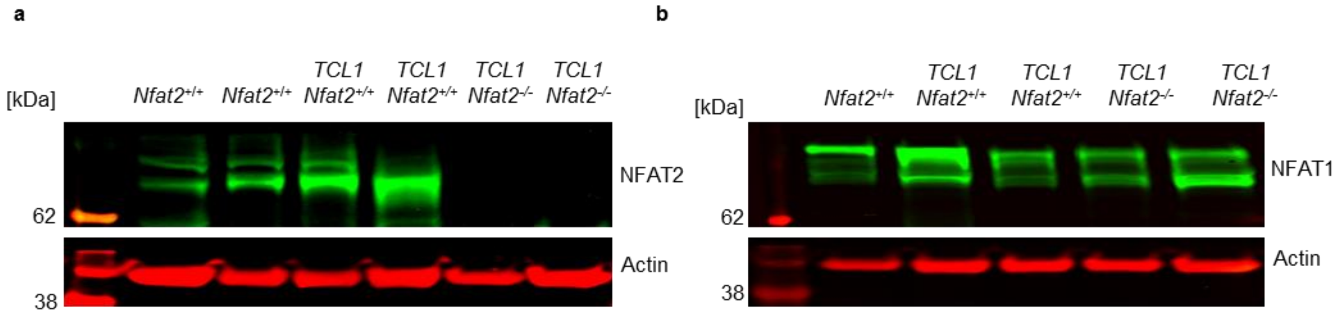


**Supplementary Figure 1: Expression of NFAT proteins in *Nfat2*-deleted B cells**

**(a+b)** Protein expression of NFAT2 **(a)** and NFAT1 **(b)** in isolated splenic B cells from WT *Nfat2*$^{+/+}$, *TCL1 Nfat2*$^{+/+}$ and *TCL1 Nfat2*$^{-/-}$ mice assessed by western blotting. Actin was used as loading control.

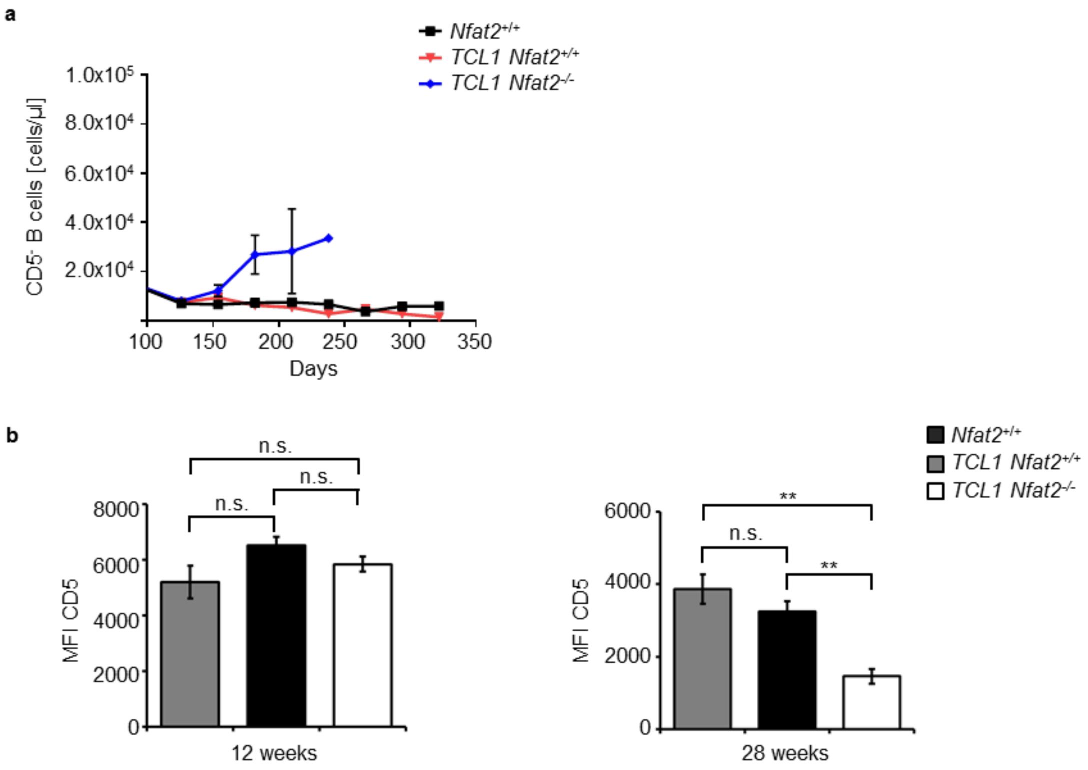


**Supplementary Figure 2: Downregulation of CD5 expression on the CLL cells of *TCL1 Nfat2*$^{-/-}$ mice**

**(a)** Expansion of CD19$^{+}$CD5$^{-}$ B cells in the peripheral blood of *Nfat2*$^{+/+}$ (n=5), *TCL1 Nfat2*$^{+/+}$ (n=10) and TCL1 *Nfat2*$^{-/-}$ (n=10) mice assessed by flow cytometry at the indicated time points.

**(b)** Mean Fluorescence intensity (MFI) of CD5 of CD19+ B cells from *Nfat2*$^{+/+}$ (n=5), *TCL Nfat2*$^{+/+}$ (n=8) and *TCL1 Nfat2*$^{-/-}$ (n=8) mice at the age of 12 weeks and 28 weeks was assessed by flow cytometry (Student's t-test, Mean ± SEM, *** $p < 0.005$, not significant (n.s.).

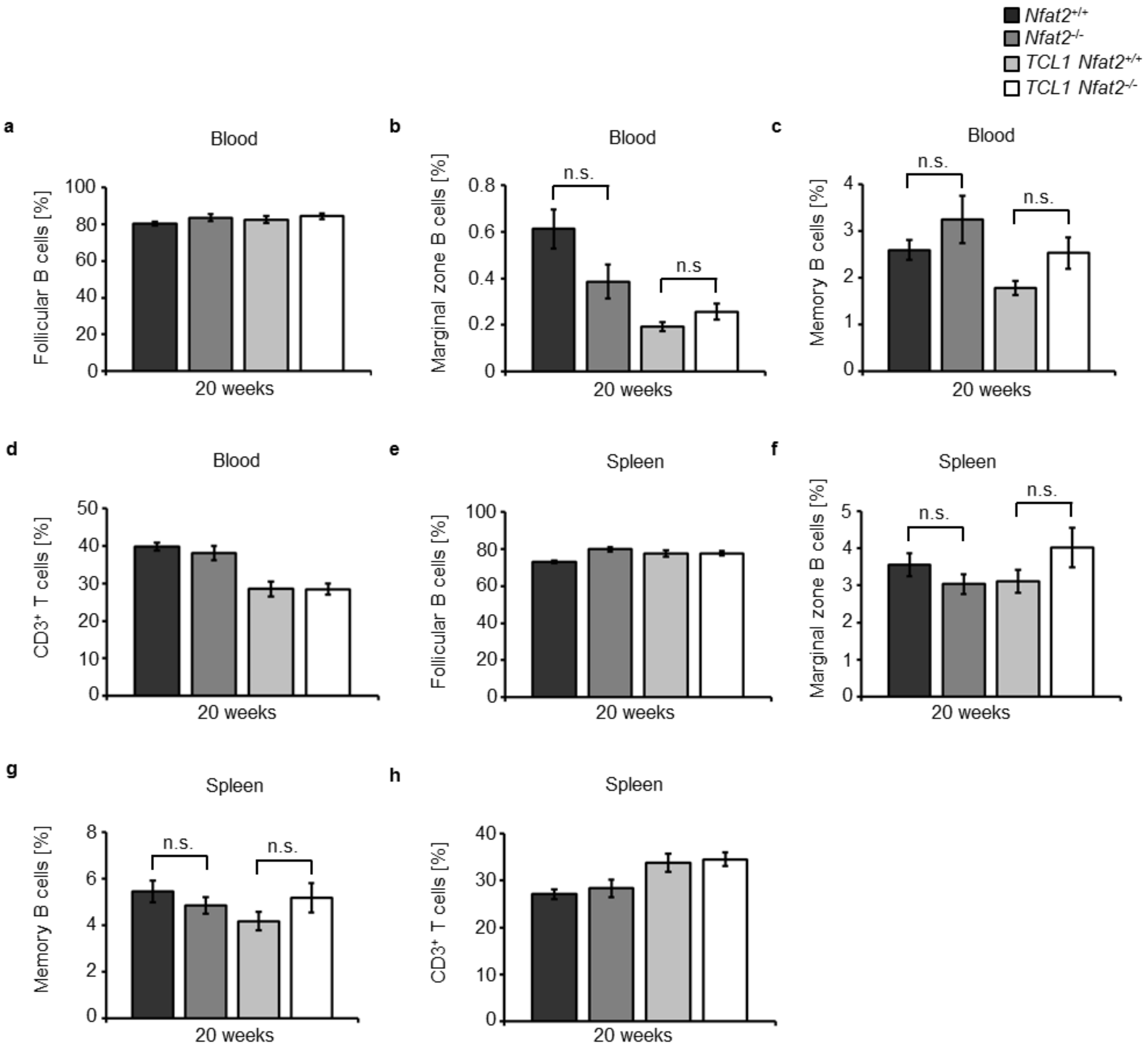


**Supplementary Figure 3: Affection of other lymphoid subpopulations by *Nfat2* deletion**

**(a-h)** Lymphoid subpopulations in the peripheral blood **(a-d)** and spleen **(e-h)**) of *Nfat2*$^{+/+}$, *Nfat2*$^{-/-}$, *TCL1 Nfat2*$^{+/+}$ and *TCL1 Nfat2*$^{-/-}$ mice (n=5 per group) at the age of 20 weeks analyzed by flow cytometry. Percentage of follicular B cells (LIN$^{-}$CD93$^{-}$B220$^{+}$CD21$^{lo}$CD23$^{+}$), marginal zone B cells (LIN$^{-}$CD93$^{-}$B220$^{+}$CD21$^{+}$CD23$^{lo\text{-}hi}$), memory B cells (LIN$^{-}$CD93$^{-}$B220$^{+}$IgD$^{-}$IgM$^{-}$), and T cells (CD3$^{+}$CD19$^{-}$) (Student's t-test, Mean ± SEM, not significant (n.s.).

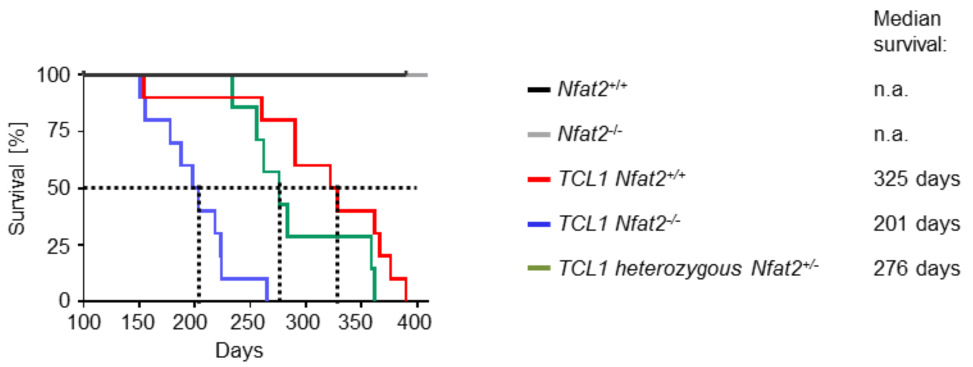


| Curve comparison: | Median survival | Log Rank test | Gehan-Breslow-Wilcoxon test |
|---|---|---|---|
| *TCL1 Nfat2*$^{+/+}$ with *TCL1 Nfat2*$^{-/-}$ | 325 vs 201 days | 0.0002 *** | 0.0011 ** |
| *TCL1 Nfat2*$^{-/-}$ with *TCL1 het. Nfat2*$^{+/-}$ | 201 vs 276 days | 0.0008 *** | 0.0014 ** |
| *TCL1 Nfat2*$^{+/+}$ with *TCL1 het. Nfat2*$^{+/-}$ | 325 vs 276 days | 0.0994 n.s. | 0.1432 n.s. |

**Supplementary Figure 4: Survival of *TCL1 Nfat2*$^{+/-}$mice**

Kaplan-Meier plot of *Nfat2*$^{+/+}$ (n=5), *Nfat2*$^{-/-}$ (n=5), *TCL1 Nfat2*$^{+/+}$ mice (n=10), *TCL1 Nfat2*$^{-/-}$ (n=10) and heterozygous *TCL1 Nfat2*$^{+/-}$ (n=10) mice. Statistical significance was determined using a Log-rank (Mantel-Cox) test and a Gehan-Breslow-Wilcoxon test, not available (n.a.).

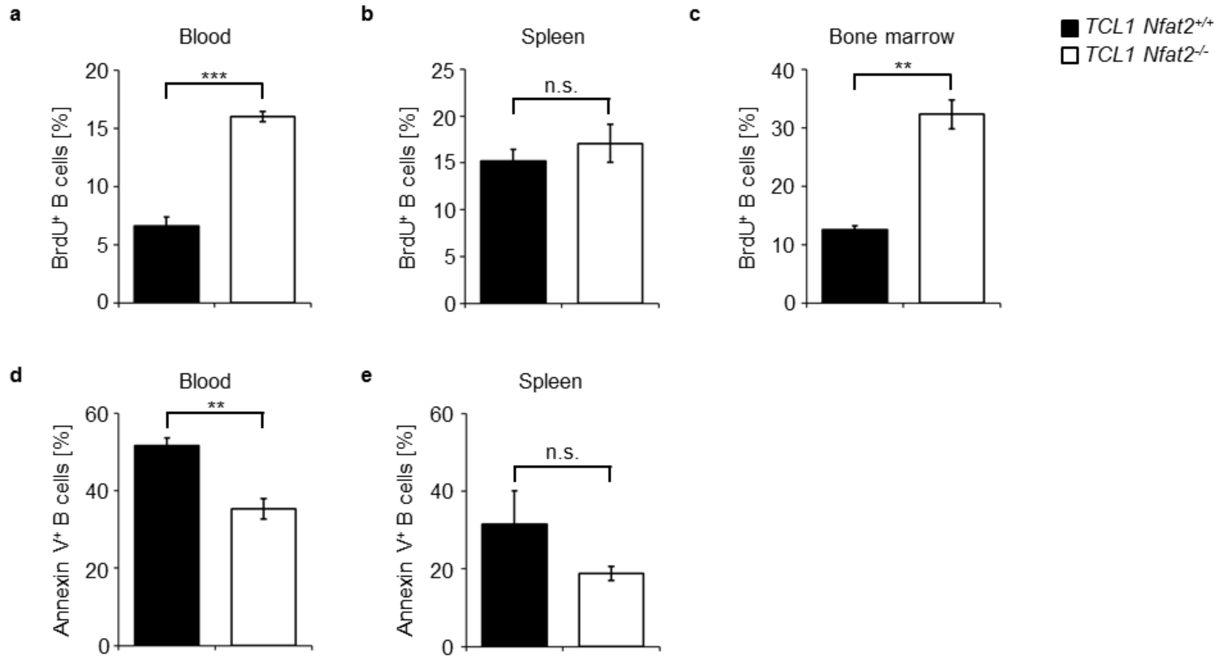


**Supplementary Figure 5: Analysis of proliferation and apoptosis of CLL cells transplanted into NSG mice**

**(a-e)** NSG mice (n=5 per group) were transplanted with CLL cells from *TCL1 Nfat2*$^{+/+}$ or *TCL1 Nfat2*$^{-/-}$ mice. 5 weeks after transplantation proliferation and apoptosis was assessed by flow cytometry. Mice were injected with 10 mM BrdU i.p. and cells were harvested after 24 h. CD19$^{+}$ B cells were stained with BrdU **(a-c)** and Annexin V antibodies **(d+e)** and measured by flow cytometry. (Student's t-test, Mean ± SEM, * p<0.05, ** p<0.01, *** p<0.005, not significant (n.s.)).

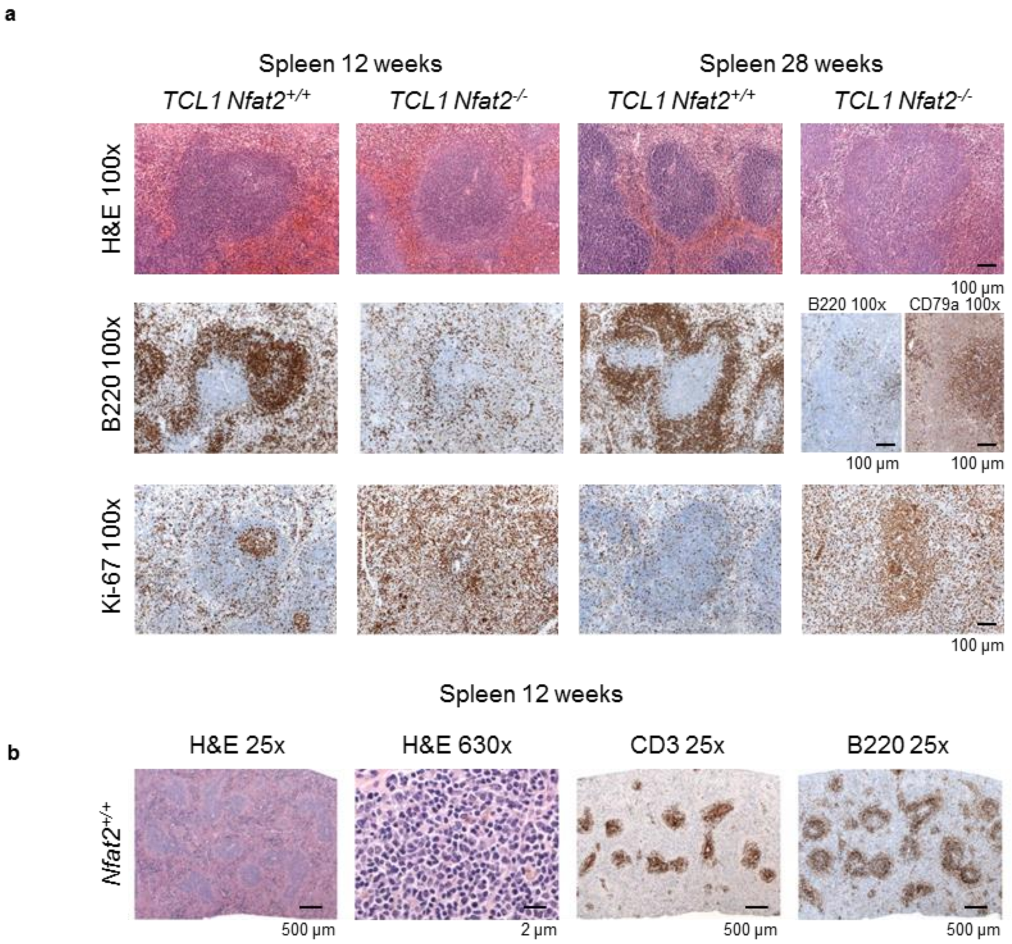


**Supplementary Figure 6: Histopathological analysis of spleen sections from *TCL1 Nfat2$^{-/-}$* mice**

**(a)** H & E staining of paraffin-embedded spleen sections of representative *TCL1 Nfat2$^{+/+}$* and *TCL1 Nfat2$^{-/-}$ m*ice at 12 and 28 weeks of age (upper panels) and immunohistochemical staining for B220, CD79a and Ki-67 (lower panels).

**(b)** H & E staining and immunohistochemical staining for CD3 and B220 of paraffin-embedded spleen sections of one representative *Nfat2$^{+/+}$* wild type mouse at 12 weeks of age.

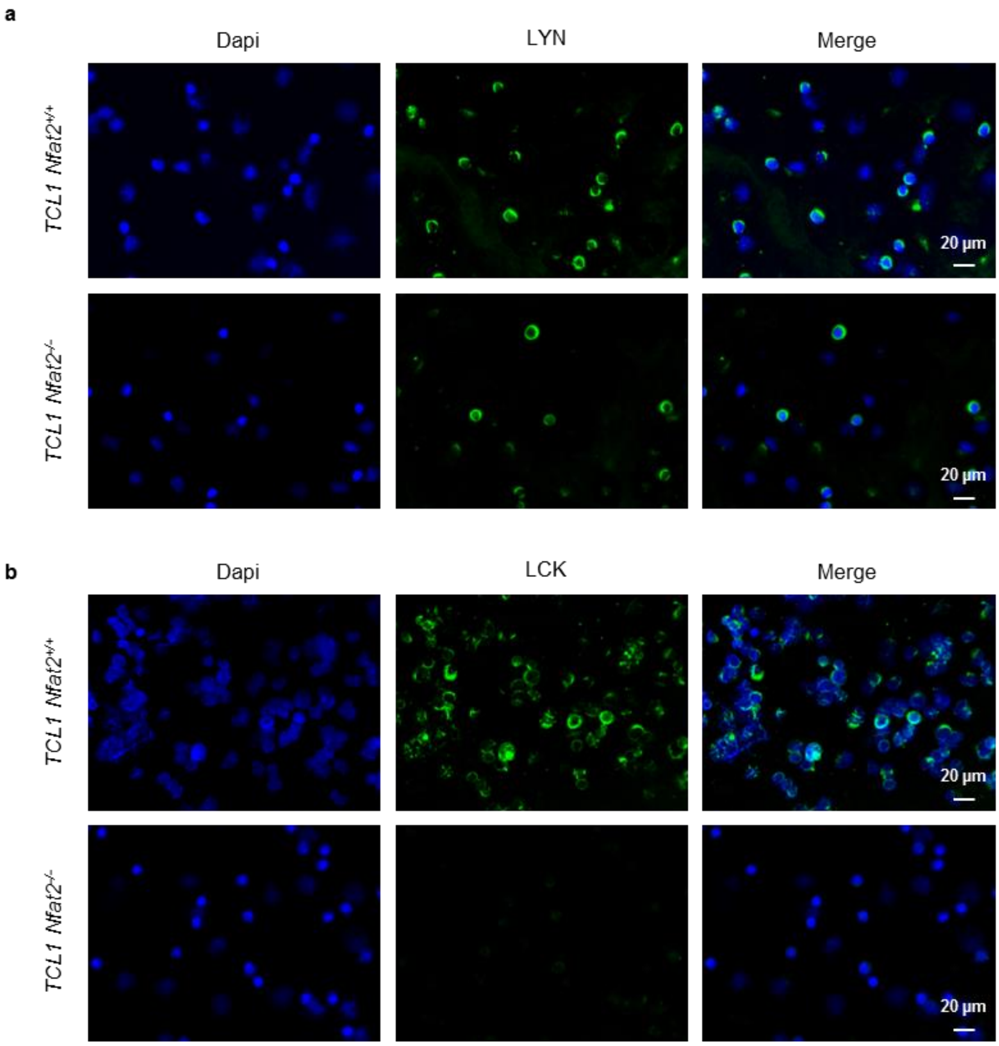


**Supplementary Figure 7: Immunohistochemical staining of CLL cells for LYN and LCK**

**(a+b)** Cytospins of CLL cells from *TCL1 Nfat2*$^{+/+}$ and *TCL1 Nfat2*$^{-/-}$ mice were prepared. Cells were stained with antibodies for LYN **(a)** or LCK **(b)**, nuclei were stained with DAPI (200x).

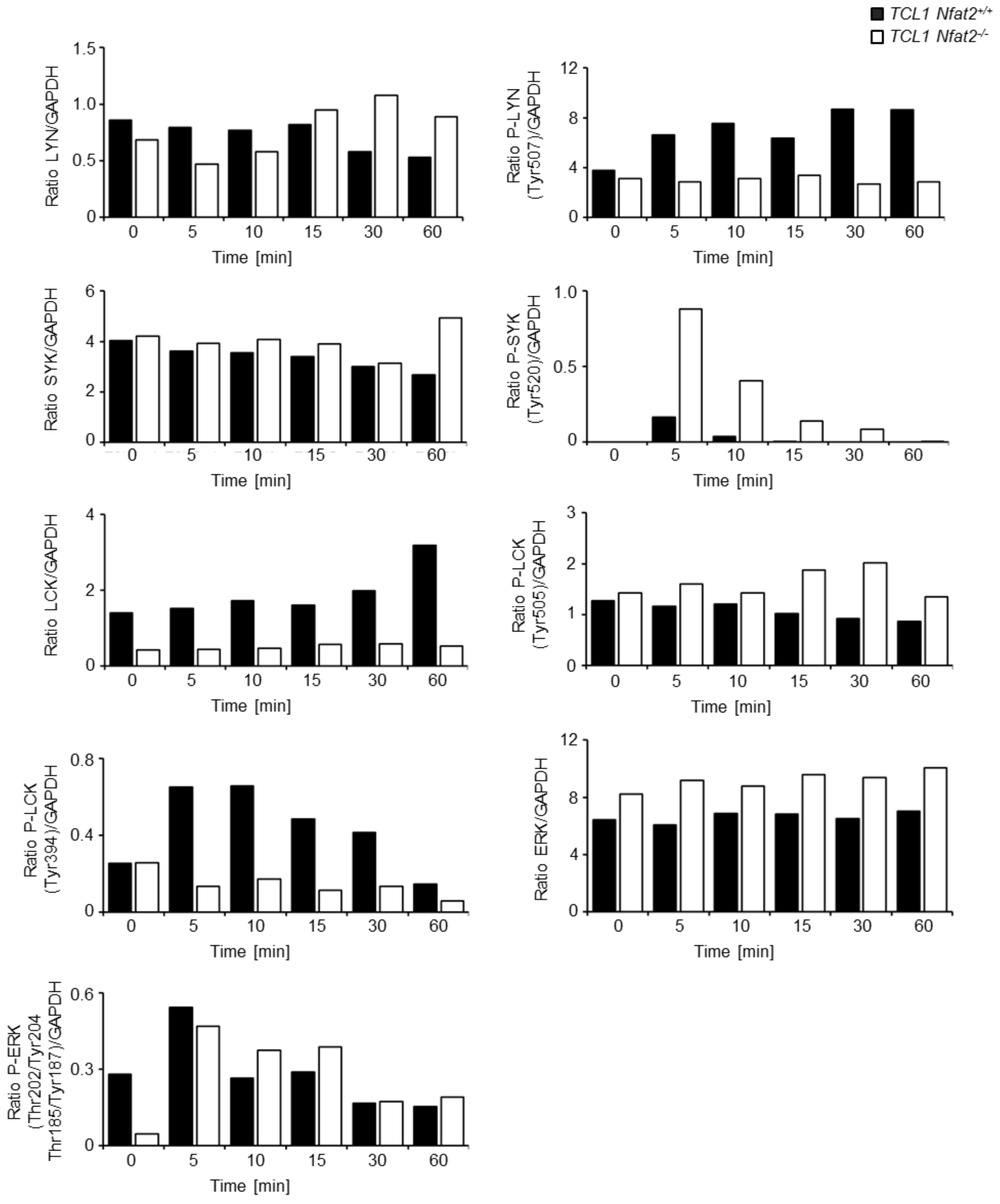


**Supplementary Figure 8: Quantitative analysis of signaling events in CLL cells**

**(a-i)** Splenic CD19$^{+}$CD5$^{+}$ CLL cells from *TCL1 Nfat2*$^{+/+}$ and *TCL1 Nfat2*$^{-/-}$ mice were stimulated *in vitro* with 20 µg/mL αIgM for the time points indicated. Total protein levels and phosphorylation status was assessed by western blotting. Visible signals were quantified with the LI-COR Odyssey Imaging software and the ratio of target protein to GAPDH is displayed. Quantification corresponds to Fig. 6d.

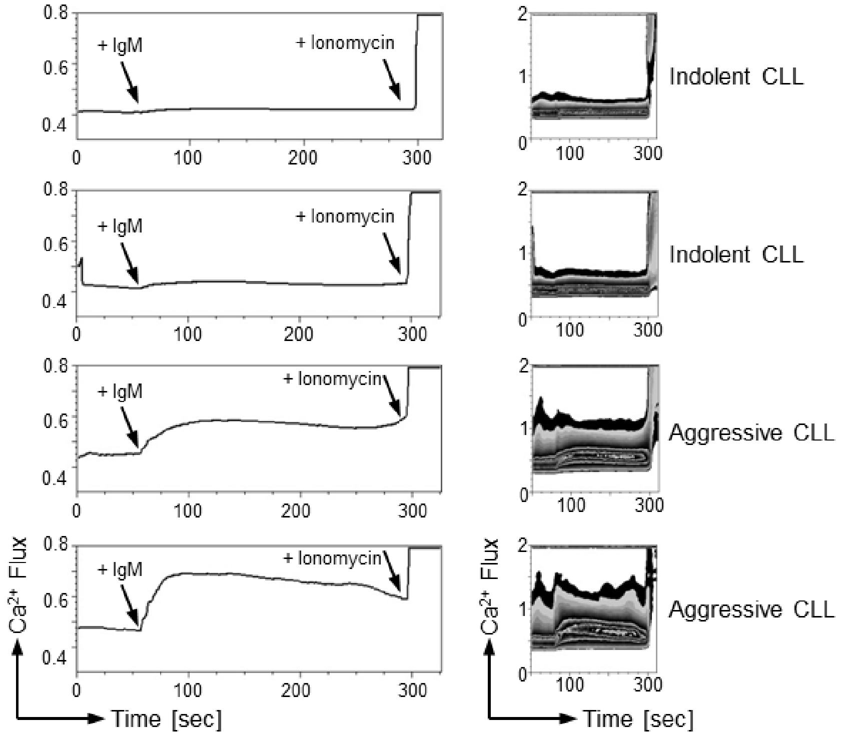


**Supplementary Figure 9: Calcium mobilization capacity of indolent and aggressive CLL**

$Ca^{2+}$ flux of $CD19^{+}$ CLLs cells of two representative indolent and aggressive CLL patients after stimulation with 20 µg/mL αIgM. 1 µM ionomycin was added as a positive control. $Ca^{2+}$ flux was calculated with the ratio of bound and unbound FuraRed and displayed with kinetics (left panel) or classical density plots (right panel).

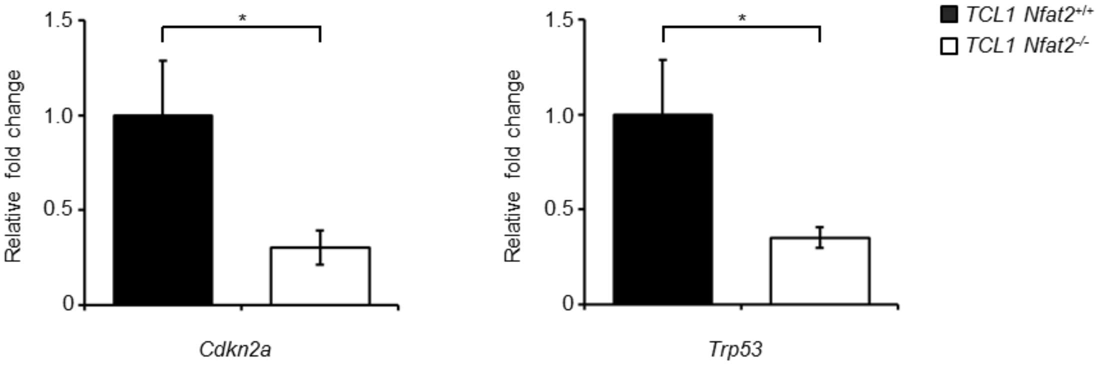


**Supplementary Figure 10: Genes associated with Richter's Syndrome are downregulated in *TCL1 Nfat2*$^{-/-}$**

Relative gene expression of *Cdkn2a and Trp53* mRNA normalized to *Actin* expression in *ex vivo* splenic CLL cells from *TCL1 Nfat2*$^{+/+}$ and *TCL1 Nfat2*$^{-/-}$ mice (n=6 per group) assessed by qRT-PCR (Student's t-test, Mean ± SEM, * $p < 0.05$).

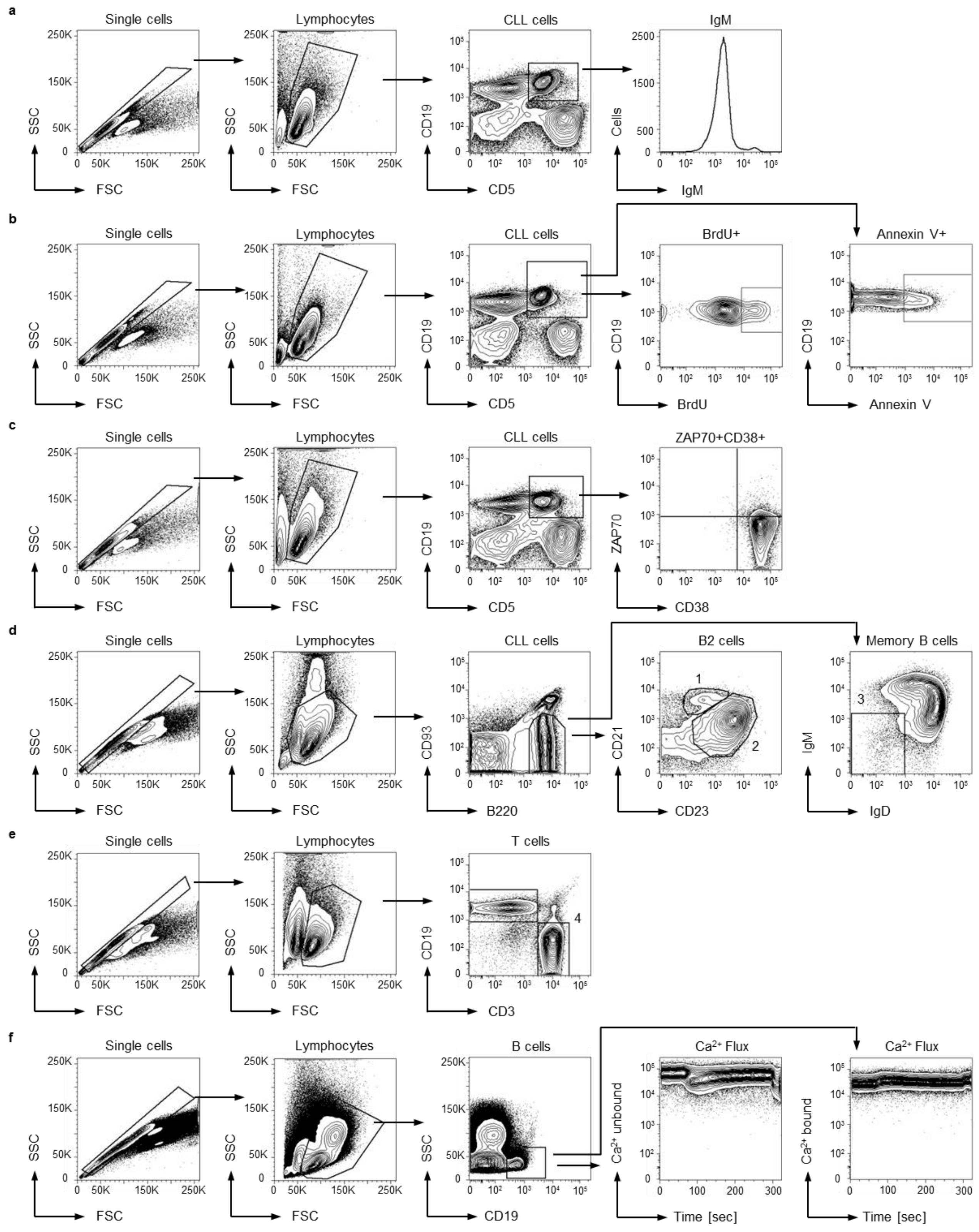


**Supplementary Figure 11: Gating strategies used for FACS analysis**

**(a)** Gating strategy for the detection of IgM surface expression in Figure 1e and Figure 7b.

**(b)** Gating strategy for the analysis of proliferation and apoptosis in Figure 2e.

**(c)** Gating strategy for the detection of ZAP70 and CD38 expression in Figure 3h.

**(d+e)** Gating strategy for the detection of different lymphoid subpopulations in Supplementary Figure 3: marginal zone B cells (1), follicular B cells (2), memory B cells (3) and T cells (4).

**(f)** Gating strategy for $Ca^{2+}$ measurements in Supplementary Fig. 9.

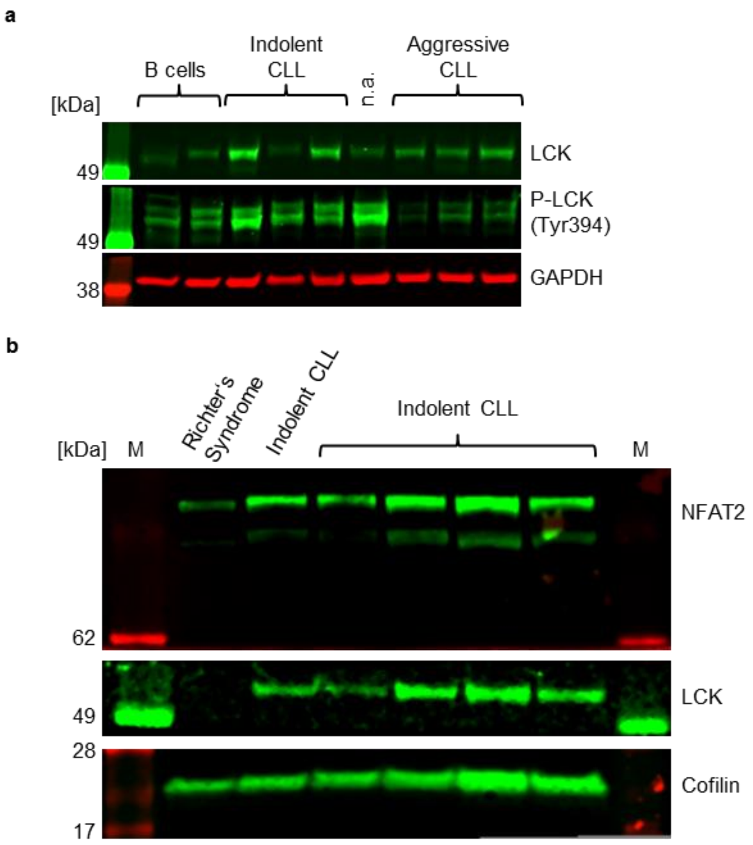


**Supplementary Figure 12: Uncropped Western Blots**

**(a)** Uncropped Western Blot related to Figure 6d showing the expression of LCK and of its activated form with an activating phosphorylation at Tyr394 in physiological B cells (n=2), indolent CLL (n=3) and aggressive CLL (n=3) cells. Sample n.a. was excluded because it could not be assigned to one of the two categories (indolent or aggressive) due to lacking patient information.

**(b)** Uncropped Western Blot related to Figure 7g showing NFAT2 and LCK protein expression in one patient with Richter's syndrome (n=1) and five patients with indolent CLL. (n=5).

| Parameter | CLL [n=24] | Indolent CLL [n=14] | Aggressive CLL [n=10] |
|---|---|---|---|
| Medium Age [years] | 69.1 | 69.0 | 69.2 |
| Sex [male/female] | 15/9 | 9/5 | 6/4 |
| Stage Binet B or C | 10 (41.6%) | 1 (10%) | 9 (90.0%) |
| Patients with at least one treatment | 12 | 2 (14%) | 10 (100 %) |
| Median number of treatments received | 3.3 | 1 | 3.8 |
| Time to first treatment < 48 months | 6 (25%) | 0 (0%) | 6 (60%) |
| IGHV mutated | 9 (37.5%) | 9 (64.2%) | 0 (0%) |
| High risk genetic aberration (del17, del11, TP53 mutation) | 3 (12.5%) | 0 (0%) | 3 (30%) |

**Supplementary Table 1: Classification of CLL patients in indolent and aggressive categories**

Classification:

Cases of indolent disease fulfilled at least 3 of the following criteria:

1. No treatment requirement for at least 7 years
2. Initial Binet Stage A
3. IGHV mutated
4. No high risk genetic aberration (TP53 mutation, del 17p, del 11q)

Cases of aggressive disease fulfilled at least 3 of the following criteria:

1. Treatment requirement within less than 4 years
2. Initial Binet Stage B or C
3. IGHV unmutated
4. Presence of high risk genetic aberration (TP53 mutation, del 17p, del 11q)

| Samples | Gene | Sequence |
|---|---|---|
| PBMC samples | *NFAT2* | FW: 5’-GGCTGCGGTCTTCGGGAGAG-3’<br>RV: 5’-AGCCATAGTGTTCTTCCTCCGCTGA-3’ |
| | *GAPDH* | FW: 5’-GGGTGTGAACCATGAGAAG-3’<br>RV: 5’-GGCAGGGATGATGTTCTGG-3’ |
| Lymph nodes | *NFAT2* | FW: 5’-ACCGAGCCCACTACGAGAC-3’<br>RV: 5’-CGGCTCATTCTCCAAGTAGC-3’ |
| | *GAPDH* | FW: 5’-ATTGCCGACAGGATGCAGAA-3’<br>RV: 5’-GTACTTGCGCTCAGGAGGAG-3’ |
| Mouse CLL cells | *Prdm1* | FW: 5’-TAGACTTCACCGATGAGGGG-3’<br>RV: 5’-GTATGCTGCCAACAACAGCA-3’ |

**Supplementary Table 2: Primers were used for qRT-PCR analysis**

Primers were used for qRT-PCR analysis of mouse CLL cells and of human PBMC or lymph node samples. Primers with shorter amplicon length were needed for paraffin embedded lymph node samples.

| Gene | Sequence |
|---|---|
| Lck-Promotor | FW: 5’-CAGGCAAAACAGGCACACAT-3’<br>RV: 5’-CCTCCAGTGACTCTGTTGGC-3’ |
| CD40L-Promotor | FW: 5’-ACTCGGTGTTAGCCAGG-3’<br>RV: 5’-GGGCTCTTGGGTGCTATTGT -3’ |

**Supplementary Table 3: Primers used for qRT-PCR analysis of ChIP samples.**
